# In Vitro Immuno-Modulatory Potentials of Purslane (*Portulaca oleracea* L.) Polysaccharides with a Chemical Selenylation

**DOI:** 10.3390/foods11010014

**Published:** 2021-12-21

**Authors:** Ya-Ru Lin, Qing-Yun Guan, Ling-Yu Li, Zhi-Mei Tang, Qiang Zhang, Xin-Huai Zhao

**Affiliations:** 1 School of Biology and Food Engineering, Guangdong University of Petrochemical Technology, Maoming 525000, China; lin17853509810@163.com (Y.-R.L.); mguan4826@163.com (Q.-Y.G.); 17852026615@163.com (L.-Y.L.); tzm1103@gdupt.edu.cn (Z.-M.T.); zhangqiang@gdupt.edu.cn (Q.Z.); 2 Research Centre of Food Nutrition and Human Healthcare, Guangdong University of Petrochemical Technology, Maoming 525000, China; 3 Maoming Branch, Guangdong Laboratory for Lingnan Modern Agriculture, Guangdong University of Petrochemical Technology, Maoming 525000, China

**Keywords:** purslane polysaccharides, selenylation, RAW 264.7 macrophages, splenocytes, immune modulation

## Abstract

The soluble polysaccharides from a non-conventional and edible plant purslane (*Portulaca oleracea* L.), namely PSPO, were prepared by the water extraction and ethanol precipitation methods in this study. The obtained PSPO were selenylated using the Na_2_SeO_3_-HNO_3_ method to successfully prepare two selenylated products, namely SePSPO-1 and SePSPO-2, with different selenylation extents. The assay results confirmed that SePSPO-1 and SePSPO-2 had respective Se contents of 753.8 and 1325.1 mg/kg, while PSPO only contained Se element about 80.6 mg/kg. The results demonstrated that SePSPO-1 and SePSPO-2 had higher immune modulation than PSPO (*p* < 0.05), when using the two immune cells (murine splenocytes and RAW 264.7 macrophages) as two cell models. Specifically, SePSPO-1 and SePSPO-2 were more active than PSPO in the macrophages, resulting in higher cell proliferation, greater macrophage phagocytosis, and higher secretion of the immune-related three cytokines, including tumor necrosis factor-α (TNF-α), interleukin-6 (IL-6), and IL-1β. Meanwhile, SePSPO-1 and SePSPO-2 were more potent than PSPO in the concanavalin A- or lipopolysaccharide-stimulated splenocytes in cell proliferation, or more able than PSPO in the splenocytes to promote interferon-γ secretion but suppress IL-4 secretion, or more capable of enhancing the ratio of T-helper (CD4^+^) cells to T-cytotoxic (CD8^+^) cells for the T lymphocytes than PSPO. Overall, the higher selenylation extent of the selenylated PSPO mostly caused higher immune modulation in the model cells, while a higher polysaccharide dose consistently led to the greater regulation effect. Thus, it is concluded that the employed chemical selenylation could be used in the chemical modification of purslane or other plant polysaccharides, when aiming to endow the polysaccharides with higher immuno-modulatory effect on the two immune cells.

## 1. Introduction

Purslane (*Portulaca oleracea* L.) is a wild plant belonging to the *Portulacaceae* family. Purslane is widely spread and popular in most areas, including China, Europe, and Mediterranean countries, and is edible but usually regarded as one of these non-conventional plants. More importantly, purslane is regarded as having many biofunctions in both medicine and food fields. It was first recorded in the Compendium of Material Medica that purslane leaves had the ability to clear evil heat and remove toxins [1]. Moreover, recent research results have indicated the emerging functional properties of purslane in the intestine, skin, nerve, respiratory, and other systems. For example, the extract of purslane leaves showed an ability to reduce the severity of colitis through regulating the immune mechanism involved in the pathogenesis of colitis [2]. Besides, it was also found that the purslane juice could protect the rat brain from the rotenone-caused neurotoxicity, as well as apoptosis, by inhibiting excessive oxidative stress [3]. Overall, purslane is considered to contain these bioactive components, including alkaloids, polysaccharides, unsaturated fatty acids, flavonoids, proteins, and others, thus being regarded with various beneficial functions, such as anti-bacterial, anti-fungal, anti-inflammatory, analgesic, muscle relaxant, and wound healing effects. In addition, purslane is unusually rich in aliphatic acids (e.g., the α-linolenic acid) that are important in cholesterol reduction and blood lipid-lowering, as well as anti-thrombotic or anti-cardiovascular effect [4], while the flavone compounds in purslane also are of importance for the vital hypoglycemic and anti-oxidative functions by inhibiting the Akt phosphorylation to enhance the consumption of glucose or by scavenging free radicals and reducing metal ions, such as Fe^3+^ [5]. Overall, the potential health benefits of purslane are still insufficiently investigated so far.

Polysaccharides, a kind of carbohydrates, are made up of more than ten monosaccharide units that are joined through the glycosidic bonds in the branched or unbranched chains. For purslane, it was reported the polysaccharides extracted by water had a molecular mass about 7.3 kDa, with arabinose, galactose, glucose, mannose, rhamnose, and xylose as main saccharide elements [6]. It is worth mentioning that natural polysaccharides have various pharmacological effects, such as anti-cancer, anti-inflammation, anti-oxidation, modulation of gut microbiota, and immune function [7]. For example, the natural polysaccharides might exert hepatoprotective effect by regulating the pathways of inflammation and apoptosis, lipid metabolism, and cytochrome P450 enzymes [8], while the acidic polysaccharides from *Schisandra chinensis*, through reducing the oxidative stress, could protect the acute liver injury induced by ethanol [9]. Additionally, the combined fungal polysaccharides could suppress the hepatotoxicity induced by cyclophosphamide through reducing toxicity markers and preventing inflammatory responses [10]. As is reported, anti-oxidation and hypoglycemic effect are two important biological functions of plant polysaccharides. It was reported that the polysaccharides from garlic (*Allium sativum* L.) bolt and green walnut (*Juglandaceae*) husk possessed anti-oxidant activity to scavenge three radicals or reduce the multi-valent metal ions, such as Fe_3_^+^ [11,12], while those from bluish dogbane (*Apocynum venetum*) leaves had anti-hypoglycemic effect in type 2 diabetes mice by regulating intestinal flora, along with reducing glucose absorption [13]. Today, cancer is one of the most fatal diseases in the world; thus, the anti-tumor activities of polysaccharides by inhibiting tumor growth and enhancing immunological functions have attracted a special attention in recent [14,15]. For example, the polysaccharides from shiitake mushrooms (*Lentinus edodes*) could exert anti-tumor activities to the colon cancer HT-29 cells via cell proliferation suppression and apoptosis induction, through an internal pathway mediated by reactive oxygen species (ROS) and external pathway engaged with TNF-α [16]. In referring purslane polysaccharides, they were reported to have anti-diabetic effect in diabetic rats and could enhance the immune state of the rats with gastric cancer [6,17].

Immune modulation of natural plant polysaccharides and other components are also sufficiently studied. The immune system is the body’s defensive system that performs the immune responses, immune function, and self-protection, and is composed of these elements, such as immune organs, cells, and molecules. The immunological responses consist of the innate and adaptive immune responses, including humoral and cellular immunity, while the responses to external stimulation are considered as one of the body’s key defending strategies to prevent and combat external infections, inflammation, and cancers [18]. Some natural substances derived from natural foods have immuno-modulatory effects. Tea polyphenols could increase the immunity of tilapia via promoting the activity and expression of immunoglobulin, enhancing the lysozyme activity, and regulating the NF-κB signaling pathway [19]. The peptides derived from whey and casein proteins had a terrific immuno-modulatory function because the peptides could increase the macrophage phagocytosis, promote splenocyte proliferation, and enhance cytokine secretion [20,21]. In addition, a flavonoid compound, quercetin, also might improve the immunity of *Arbor Acre* broilers [22]. Overall, it was revealed that plant polysaccharides could play an effective immuno-modulatory role in immune systems through activating the macrophages, splenocytes, and other immune cells, promoting the release of cytokines, increasing the growth of immune organs and the secretion of immunoglobulins, and inhibiting the over-activation of the complement system [23]. However, whether a chemical modification of natural polysaccharides will cause positive or negative effects on the immune modulation of the modified polysaccharides is less studied in the present. Thus, such an investigation using the soluble purslane polysaccharides as a target for a chemical selenylation deserves our consideration.

In this study, the soluble polysaccharides from purslane (namely PSPO) were extracted by water at a neutral condition, and then selenylated chemically using the Na_2_SeO_3_-HNO_3_ system for two selenylation extents to prepare two selenylated PSPO products (SePSPO), namely SePSPO-1 and SePSPO-2, respectively. Both SePSPO-1 and SePSPO-2 were assessed for their in vitro immuno-modulatory activities using two immune cells (i.e., the RAW 264.7 macrophages and murine splenocytes) as cell models and the unmodified PSPO as a control. Several indices, such as growth proliferation, phagocytic activity, cellular secretion of five cytokines, and T lymphocyte subpopulations, were measured and compared to reflect the target immune modulation. The purpose of this study was to disclose whether the performed chemical selenylation could cause bioactivity changes for the soluble PSPO in their important immune potential.

## 2. Materials and Methods

### 2.1. Materials and Reagents

The RPMI-1640 medium, Dulbecco’s modified essential medium with high glucose (DMEM) were purchased from HyClone Co. (Logan, UT, USA), while the fetal bovine serum (FBS) was provided by Thermo Fisher Scientific Inc. (Cleveland, OH, USA). Both neutral red and trypan blue were purchased from Amresco Inc. (Los Angeles, CA, USA), and 3-(4,5-dimethyl-2-thiazolyl)-2,5-diphenyl tetrazolium bromide (MTT), concanavalin A (ConA), and lipopolysaccharide (LPS) were provided by Sigma-Aldrich Chemical Co. (St. Louis, MO, USA). The phosphate-buffered saline (PBS) was the product of Solarbio Science and Technology Co. Ltd. (Beijing, China), while the Hanks’ balanced salt solution (HBSS) and the red blood cell lysis buffer were obtained from Beyotime Biotechnology (Beijing, China). The cell counting kit-8 (CCK-8) was the product of Dojindo Laboratories (Kyushu, Japan). Ultrapure water generated from Milli-Q Plus (Millipore Corporation, New York, NY, USA) was used in this study. Other chemicals used in the present work were of analytical grade.

The phycoerythrin (PE)-conjugated anti-mouse CD8a^+^ antibodies and fluorescein isothiocyanate (FITC) anti-mouse CD4^+^ antibodies were bought from Miltenyi Biological Technology Co. Ltd. (Bergisch Gladbach, Cologne, Germany), while the enzyme-linked immunosorbent assay (ELISA) kits [mouse interferon-γ (IFN-γ), interleukin-1β (IL-1β), IL-4, IL-6, and tumor necrosis factor-α (TNF-α)] were bought from Boster Biological Engineering Co. Ltd. (Wuhan, China).

### 2.2. Animal and Cells

The used mice (female BALB/c, 6–8 weeks old) in this study were provided by professional institution Beijing Vital River Experimental Animal Technical Co. Ltd. (Beijing, China). As usual, the mice were maintained for at least 7 d before the experiments performed at Northeast Agricultural University (Harbin, China). In addition, all animal procedures were approved and instructed by Animal Care and Use Committee of Northeast Agricultural University.

The used RAW 264.7 macrophages, provided by Shanghai Branch of Chinese Academy of Sciences (Shanghai, China), were incubated in the DMEM medium supplemented with 10% fetal bovine serum (FBS) and 100 U/mL streptomycin/penicillin. The cells were cultured in an incubator of 37 °C and 5% CO_2_ referring to the recommendation of cell supplier.

### 2.3. Polysaccharide Extraction

The water-extraction and ethanol-precipitation protocols were used to extract PSPO as previously described with minor modification [24,25]. In detail, the dried purslane materials were smashed into powder, blended with water at a 1:20 ratio (*w*/*v*), added with the thermostable α-amylase of 20 U/mL, and kept at 90 °C for 4 h, followed by a centrifugation at 8000× *g* for 15 min after cooling. Afterwards, the separated supernatant was filtered and mixed with an alkaline protease (100 U/mL) at 55 °C for 8 h to degrade the extracted proteins, and then centrifuged once again at 8000× *g* for 15 min after cooling. The obtained supernatant was concentrated to 1/10 of the original volume by heating and then precipitated by using anhydrous ethanol of three-fold volume at 4 °C for 12 h. The precipitates (i.e., PSPO) were separated by using an 8000× *g* centrifugation for 15 min, washed three times using anhydrous ethanol, and soaked in anhydrous ethyl ether to remove fats and pigments. After that, the PSPO was dialyzed against water for 2 d to remove the small-molecule impurities and salt ions, freeze-dried, and then kept at −20 °C for future usage.

### 2.4. PSPO Selenylation and Se Detection

The Na_2_SeO_3_-HNO_3_ method was applied to prepare selenylated PSPO according to the previous study [26]. Briefly, 300 mg PSPO powder was dissolved in 20 mL 5% HNO_3_, mixed with 30 or 45 mg Na_2_SeO_3_ and reacted at 75 °C for 8 h. Three-fold volume of anhydrous ethanol was added into the reaction mixture after cooling, while the final system was kept at 4 °C for 12 h. Thus, the precipitates were collected, soaked in anhydrous ethanol five times to remove the unreacted H_2_SeO_3_, and then freeze-dried to obtain two selenylated products, namely SePSPO-1 and SePSPO-2, respectively. Besides, the same amount of PSPO was mixed with Na_2_SeO_3_ without HNO_3_ and subjected to the same treatments. The yielded PSPO were regarded as the unmodified PSPO and used as a control in this study.

Se contents of the target samples were assessed by the method reported in a previous study [27], using an inductively coupled plasma-mass spectrometer (Agilent Technologies, Santa Clara, CA, USA).

### 2.5. Assays of Cell Viability and Phagocytic Activity of the Macrophages

The possible effects of PSPO, SePSPO-1, and SePSPO-2 on macrophage viability were measured by a MTT assay, as previously described [28]. Specifically, 100 μL cells (2 × 10^5^ cells/mL) were inoculated into a 96-well plate, followed by 4 h culture. After medium discarding, the adherent cells were exposed to the target samples at 5–80 μg/mL for 24 or 48 h. After removing the supernatants, 100 μL MTT solution was added, and the macrophages were cultured for 4 h. After discarding the supernatants, DMSO of 100 μL was added into each well, while the optical density (OD) value of each well was determined at 450 nm with a microplate reader (Bio-Rad Laboratories, Hercules, CA, USA). The value of macrophage viability was calculated accordingly [29]. Meanwhile, the macrophages of control group without sample treatment were considered with 100% cell viability.

Macrophage phagocytosis was measured by a neutral red assay as a previous study did [30]. In detail, 200 μL cells (2 × 10^5^ cells/mL) were seeded into 96-well plates and cultured for 4 h. The target samples at doses of 5–20 μg/mL were added, while the macrophages were cultured for another 24 h. The samples were replaced by 1% neutral red solution of 100 μL, while the macrophages were incubated for 1.5 h and washed for five times by the PBS (0.1 mmol/L, pH 7.2). The cell lysing solution (ethanol: 1% acetic acid = 1:1, *v*/*v*) of 200 μL was added into each well, and the macrophages were incubated for another 2 h. The OD values were measured at 540 nm after this incubation at the same microplate reader. Phagocytic index (PI) reflecting the target macrophage phagocytosis was, thus, calculated accordingly [31].

### 2.6. Assays of Proliferation of the Polysaccharide Samples on Murine Splenocytes

The target splenocytes were obtained from the mice, based on the procedure reported in a previous study [32]. The mice were cervical dislocated and followed by a soak (5 min) in 75% ethanol solution. Thus, the spleens were taken out under an aseptic condition, ground into small pieces in a container with the cold HBSS, and then sifted through a sieve of 200-mesh to obtain the murine splenocytes. The collected splenocytes were cleaned by the PBS, centrifuged at 170× *g* for 5 min, suspended in 5 mL lysis buffer for 3 min, centrifuged and cleaned twice by the PBS, and resuspended in the RPMI-1640 medium fortified with 100 U/mL streptomycin/penicillin and 10% FBS. The splenocytes with a measured viability value more than 98%, adjusted into a fixed cell density of 1 × 10^6^ cells/mL, and then used in this study.

Proliferative effects of the samples on the LPS- or Con A-induced splenocytes were assayed using the CCK-8 method [33]. The splenocytes of 100 μL were plated in 96-well plates and cultured with the target samples at doses of 5–80 μg/mL for 48 h, together with LPS (10 μg/mL) or Con A (5 μg/mL), while the splenocytes incubated with the medium alone and mitogen in the medium were regarded as respective blank and control groups. After discarding the supernatants, the splenocytes were incubated with CCK-8 solutions of 100 μL for 4 h, while the OD values were detected at 450 nm using the same microplate reader.

### 2.7. Assays of Cytokine Secretion in the Macrophages and Murine Splenocytes

In brief, 2 mL macrophages (2 × 10^5^ cells/mL) were cultured in a 6-well plate for 4 h. After discarding the medium, the target samples at doses of 5–20 μg/mL were added to treat the cells for 24 h. In addition, then, a centrifugation at 500× *g* of 20 min was conducted to collect the supernatants, while the levels of TNF-α, IL-1β, and IL-6 in the supernatants were assayed in accordance with the instructions of the corresponding ELISA kits.

Secretion levels of IL-4 and IL-1β in the splenocytes were assayed using the respective the ELISA kits. Briefly, the splenocytes of 1 mL were incubated with the polysaccharide samples (doses of 5–20 μg/mL) of 0.5 mL in 12-well plates for 48 h. After that, the supernatants were collected using the centrifugation as above, while IL-4 and IL-1β levels were detected by the respective ELISA kits and the suggested protocols.

### 2.8. Assays of T Lymphocyte Subpopulations

The T lymphocyte subpopulations were analyzed by a flow cytometry protocol according to a previous study [34]. Briefly, the splenocytes (1.5 mL) were plated in 12-well plates, co-cultured with the three polysaccharide samples of 5–20 μg/mL and Con A of 5 μg/mL for 48 h. The cells were collected via a centrifugation at 170× *g* for 5 min, washed twice by the PBS, resuspended in 500 μL PBS, and then adjusted to 1 × 10^6^ cells. The prepared cell suspensions were mixed with FITC-conjugated anti-mouse CD4^+^ antibody (or PE-conjugated anti-mouse CD8a^+^ antibody) of 10 μL, kept at 4 °C for 30 min, passed through a sieve of 300-mesh, and then detected at a flow cytometry (Type BD FACS Aria II, BD Bioscience, Franklin Lakes, NJ, USA).

### 2.9. Statistical Analysis

All data reported in this study were obtained from three independent experiments or assays and expressed as means values ± standard deviations. The one-way ANOVA analysis with Duncan’s multiple range tests was used to measure the differences among the mean values, while the *p* < 0.05 was deemed to significant difference. The statistical analysis was conducted using the software SPSS version 16.0 (SPSS, Inc., Chicago, IL, USA).

## 3. Results

### 3.1. Macrophage Proliferation and Phagocytosis as Affected by the PSPO and SePSPO

Determined by the classic phenol-H_2_SO_4_ assaying method, the obtained PSPO were detected with a total saccharide content of 855.4 g/kg and ash content of 91.7 g/kg (dry basis). After the performed chemical selenylation, the obtained SePSPO-1 and SePSPO-2 were detected with Se contents of 753.8 and 1325.1 mg/kg (dry basis), respectively, while the unmodified (i.e., control) PSPO only contained Se of 80.6 mg/kg (dry basis). Compared with the control PSPO, both SePSPO-1 and SePSPO-2 had near 8-fold and 15-fold increases in Se contents. These data confirmed that both SePSPO-1 and SePSPO-2 successfully obtained a chemical selenylation, and Se element (in the status of H_2_SeO_3_) was covalently bound into the molecules of PSPO. Because PSPO, SePSPO-1, and SePSPO-2 were obviously different in Se contents, this study thereby assessed whether the performed chemical selenylation, as well as the selenylation extent, could affect the immune activity of the target PSPO.

**Figure 1:**
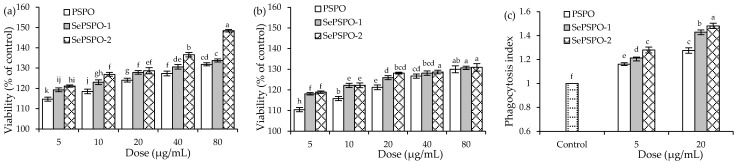
The measured cell viability values (%) of the macrophages exposed to PSPO, SePSPO-1, and SePSPO-2 for 24 (**a**) and 48 h (**b**), as well as the phagocytosis (**c**) of the macrophages exposed to PSPO, SePSPO-1, and SePSPO-2 for 24 h. Different letters lowercase above the columns suggest that the one-way ANOVA of the mean values is significantly different (*p* < 0.05).

When the three polysaccharide samples were used to treat the macrophages for 24 and 48 h using the five doses (5–80 μg/mL), the data indicated that the target samples all had no cytotoxicity on the cells (*p* > 0.05) (Figure 1a,b) because the treated macrophages showed viability values larger than 100%. With the cell treatment of 24 h, the cells exposed to PSPO, SePSPO-1, and SePSPO-2 showed viability values of 114.7–131.8%, 119.3–133.8%, and 121.1–148.4%, respectively. Using a longer time (48 h) to treat the macrophages, the cells exposed to PSPO, SePSPO-1, and SePSPO-2 showed corresponding viability values of 110.4–129.9%, 118.1–130.7%, and 118.9–130.9%. These data suggested that the samples could promote cell growth. In addition, it was observed that SePSPO-1, and especially SePSPO-2, in all cases could cause higher viability values than PSPO did, suggesting that the performed chemical selenylation resulted in higher bioactivity for PSPO, while higher selenylation extent consistently induced more bioactivity increase. Moreover, it was estimated that the SePSPO-2 dose at 20 μg/mL equaled to a Se intake near 170 μg in the body, considering a well-accepted body fluid volume of 6.4 L. This estimated Se intake is in the range of the recommended daily intake (RDI) value of Se element (50–200 μg) for humans [35]. Thus, two polysaccharide doses of 5 and 20 μg/mL were employed in the later experimental assays to obtain the secretion levels of several cytokines in the macrophages or splenocytes.

The detected phagocytic activities of the macrophages treated by the target samples were reflected in Figure 1c. The control cells had PI value of 1.00. When the cells were exposed to the samples at the doses of 5 and 20 μg/mL for 24 h, they were detected with increased PI values ranging from 1.16 to 1.48, demonstrating a fact that the samples all could enhance macrophage phagocytosis dose-dependently. Overall, PSPO and SePSPO-2 showed the respective lowest and highest potentials to promote macrophage phagocytosis, indicating again that the conducted chemical selenylation caused higher bioactivity for the PSPO, while higher selenylation extent consistently led to more activity enhancement.

### 3.2. Cytokine Secretion of the Macrophages as Affected by the PSPO and SePSPO

The secretion levels of three cytokines in the macrophages with or without the sample treatments of 24 h are shown in Table 1, using the three cytokines, including IL-6, IL-1β, and TNF-α, as three evaluation indices. In general, each sample had clear immune promotion on the cells by enhancing the secretion of the cytokines dose-dependently. The control cells had IL-6 level of 10.69 pg/mL, together with respective IL-1β and TNF-α levels of 1.55 and 1.39 pg/mL. Meanwhile, the cells treated by the samples showed much enhanced secretion in IL-6 (13.85–47.58 pg/mL), IL-1β (2.87–11.71 pg/mL), and TNF-α (68.78–144.16 pg/mL) (*p* < 0.05), while a higher polysaccharide dose consistently caused higher cytokine secretion (*p* < 0.05). Data comparison results also demonstrated that PSPO and SePSPO-2 had the respective lowest and highest capacity in the cells to elevate cytokine secretion. That is, the performed chemical selenylation endowed PSPO with a higher ability to elevate cytokine secretions in the treated cells, while higher selenylation extent also caused higher activity for the selenylated PSPO.

### 3.3. Splenocyte Proliferation as Affected by the PSPO and SePSPO

**Figure 2:**
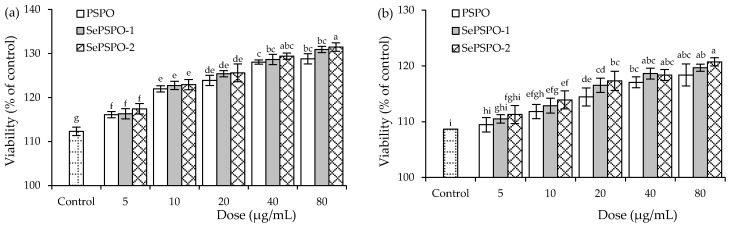
The measured viability values (%) of the splenocytes incubated with PSPO, SePSPO-1, and SePSPO-2 for 48 h and stimulated by Con A (**a**) or LPS (**b**). Different letters lowercase above the columns suggest that the one-way ANOVA of the mean values is significantly different (*p* < 0.05).

When the target samples were used at the five doses (5–80 μg/mL) with the mitogen to treat the murine splenocytes for 48 h, the treated cells all showed viability values larger than 100% (Figure 2), indicating that the samples had none cytotoxicity on the splenocytes. When the cells were stimulated by Con A, the cells exposed to PSPO, SePSPO-1, and SePSPO-2 showed viability values of 116.1–128.8%, 116.3–130.9%, and 117.4–148.4%, respectively, while the cells stimulated with Con A only had viability value of 112.3% (Figure 2a). Thus, the data suggested that higher polysaccharide doses mostly lead to larger viability values; however, the conducted chemical selenylation and yielded selenylation extent only had a minor effect on the measured viability values, although SePSPO-2 (or SePSPO-1) was slightly active than SePSPO-1 (or PSPO) to increase cell viability. When LPS was also used to stimulate the cells, a similar phenomenon was observed for the measured viability values (Figure 2b). Thus, the target samples were regarded to have immune modulation by promoting the proliferation of the mitogen-stimulated splenocytes, while the used selenylation was not sufficient to enhance PSPO bioactivity towards the stimulated cells.

### 3.4. Cytokine Secretion of the Murine Splenocytes as Affected by the PSPO and SePSPO

**Figure 3:**
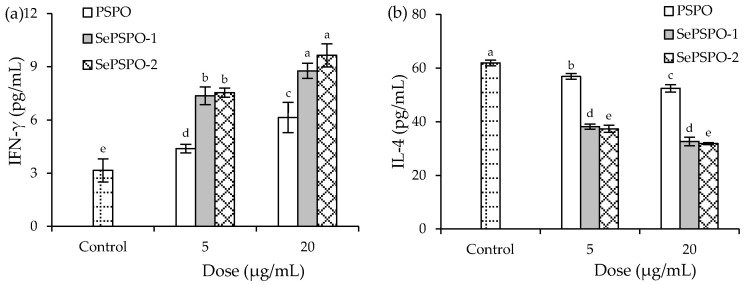
The detected levels of IFN-γ (**a**) and IL-4 (**b**) in the murine splenocytes incubated with PSPO, SePSPO-1, and SePSPO-2 for 48 h. Different letters lowercase above the columns suggest that the one-way ANOVA of the mean values is significantly different (*p* < 0.05).

The secretion levels of two cytokines IFN-γ and IL-4 in the splenocytes responding to the sample treatment of 48 h are reflected in Figure 3. Overall, each sample, in most cases, exerted a significant immune promotion in the cells by increasing the secretion of IFN-γ but clearly decreasing the secretion of IL-4. In detail, the control cells without sample treatment had IFN-γ and IL-4 levels of 3.16 and 61.91 pg/mL, respectively. When the cells were treated by PSPO, SePSPO-1, and SePSPO-2, they were measured with increased IFN-γ secretion levels (4.38–9.24 pg/mL) but distinctly decreased IL-4 secretion levels (56.88–31.90 pg/mL) (*p* < 0.05). Higher sample doses consistently caused higher secretion of IFN-γ but lower secretion of IL-4, while SePSPO-1 and SePSPO-2 were more able than PSPO to modulate the secretion of IFN-γ and IL-4. Although SePSPO-1 and SePSPO-2 had similar ability to enhance IFN-γ section, SePSPO-2 was more able than SePSPO-1 to suppress IL-4 secretion. This fact demonstrated that SePSPO-2 had higher activity in the cells than SePSPO-1. Thus, the conducted chemical selenylation conferred the PSPO with higher immune modulation in the splenocytes via regulating cytokine secretion, while higher selenylation extent generally endowed the selenylated PSPO with higher immune activity.

### 3.5. T lymphocyte Subpopulations as Affected by the PSPO and SePSPO

**Figure 4:**
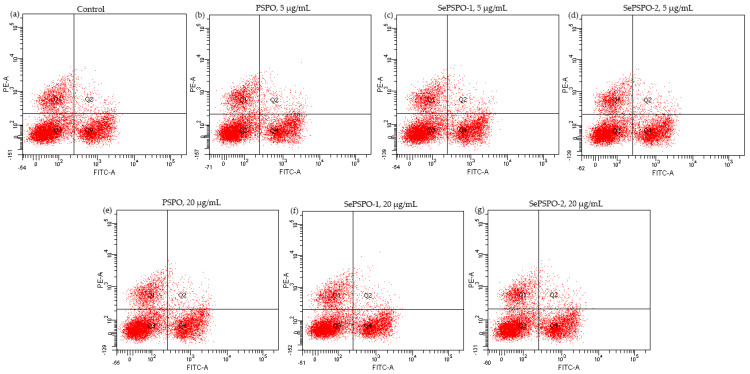
The obtained flow cytometry pictures for the control cells (**a**), and those cells incubated with PSPO (**b**), (**e**), SePSPO-1 (**c**), (**f**), and SePSPO-2 (**d**), (**g**) at the doses of 5 and 20 μg/mL, respectively.

When the splenocytes were stimulated by Con A and then treated with the target samples at the two doses of 5 and 20 μg/mL for 48 h, cell proportions of T-helper (CD4^+^) and T-cytotoxic (CD8^+^) cells were assayed using the flow cytometry technique (Figure 4). The final results are listed in Table 2, while the calculated CD4^+^/CD8^+^ ratios are also given in this table. The results showed that the control cells had a CD4^+^/CD8^+^ ratio of 2.06, while those treated by the target samples possessed enhanced CD4^+^/CD8^+^ ratios ranging from 2.08 to 2.41. Clearly, PSPO and SePSPO-2 showed the respective lowest and highest capacity to enhance the target CD4^+^/CD8^+^ ratio in the T lymphocytes. Thus, it was confirmed again that the conducted selenylation caused higher bioactivity for the selenylated PSPO, while a higher selenylation extent would make a contribution to activity enhancement.

## 4. Discussion

Conventional food or agricultural products rich in bioactive substances have been scientifically investigated for their health benefits to the body. It was reported that the acid-soluble pectin isolated from edible okra (*Abelmoschus esculentus*) had both anti-inflammatory and anti-oxidant potentials, and thereby showed a capacity to reduce NO formation and inflammatory cytokines secretion in the LPS-treated RAW 264.7 macrophages [36]. Meanwhile, the pectin from okra stem had the anti-fatigue activity by increasing both blood glucose and glycogen levels in the body [37]. In addition, the okra polysaccharides had anti-inflammation via inhibiting the phosphorylation of IκB and p65 proteins and reducing the secretion of inflammatory cytokines in the LPS-treated RAW 264.7 macrophages [38,39]. Mulberry (*Morus atropurpurea*) fruits were regarded to have beneficial biofunctions [40]. For example, the polysaccharides from black mulberry could reduce ROS formation, improve mitochondrial function, and activate the Nrf2 signaling pathway [41], and the extracts from mulberry fruits showed the anti-cancer effect on the HepG2 cells because they were capable of suppressing cell growth and inducing cell apoptosis [42]. Yam (*Dioscorea Opposita Thunb.*), cultivated widely as food and medicinal materials in China, was also considered to possess various bioactivities [43]. It was revealed in the past studies that yam polysaccharides could ameliorate insulin resistance, decrease the contents of both low-density lipoproteins and total cholesterol [44], enhance the immune functions of the lymphocytes and macrophages [45], or exert anti-tumor effect on a B16 mouse melanoma model or anti-oxidant effect on the diabetic mice [46]. At the same time, special attention has been paid to some non-conventional and edible plants to reveal their health benefits. Hawthorn (*Crataegus pinnatifida*) is known as traditional Chinese medicine, and is also a non-conventional and edible plant. These bioactive components in hawthorn, such as flavonoids and terpenoids, were revealed to have physiological functions in the cardiovascular, digestive, and endocrine systems [47]. It was also found that hawthorn polyphenols could reduce the risks of diabetes [48], or display an immuno-modulatory effect on the lymphocytes [49]. In this study, the purslane polysaccharides and two selenylated products were clarified with a clear immune modulation on the two model cells. Thus, the obtained results in this study were consistent with those results mentioned in these studies, demonstrating a beneficial function of purslane or its components to humans.

Immune modulation is one of the most fundamental biological activities of food components, considering that foodborne toxins and pathogens have various adverse effects in the body [50]. Several bioactive substances, especially those from food materials, such as polysaccharides, curcumin, and bovine lactoferrin, are considered capable of regulating or activating the immune system. Thus, various indices are used to clarify the potential immune modulation of various bioactive substances. For example, ROS formation was applied as an evaluation index [51], when aiming to identify the immune activities of polyphenol curcumin. When assessing the immuno-modulatory potentials of the target lily polysaccharides, both macrophage phagocytosis and NO content were evaluated [29]. Cytokines are the proteins essential to the cellular immunity and, thus, used widely in immune evaluation as the critical indices. For example, the lily polysaccharides and curcumin were found able to increase the secretion levels of IL-6, IL-1β, and TNF-α in the RAW 264.7 macrophages, thereby being suggested as having immuno-modulatory function [29,52]. Moreover, due to their ability to promote splenocyte proliferation, enhance the secretion levels of IL-2 and IFN-γ, and regulate T lymphocyte subpopulations, the polysaccharides from raspberry (*Rubus chingii* Hu) fruits were revealed with immune modulation in the murine splenocytes [53]. In addition, to assess the immune potential of a Zn-fortified bovine lactoferrin, the ratio of CD4^+^/CD8^+^ in the lymphocytes was used as an index [33]. In consistence with these mentioned studies, this study thereby employed these indices, such as growth proliferation, phagocytic activity, cytokine secretion, and T lymphocyte subpopulations, to evaluate the different immuno-modulatory activities of the purslane polysaccharides and two selenylated products, and, subsequently, to reveal whether the performed chemical selenylation, as well as the obtained selenylation extent, had impact on the immune function of the purslane polysaccharides. Additionally, it was found that the selenylated polysaccharides from *Artemisia sphaerocephala* exerted an increased immuno-modulation on the macrophages by promoting the expression of p-p38, p-JNK1/2, and p-ERK1/2 significantly [54], while those from *Hericium erinaceus* showed higher immune activity to the bone marrow-derived dendritic cells via activating both MAPK and NF-κB signaling pathways [55]. Thereby, it is suggested that how the target samples exerted the immuno-modulation in the two immune cells should be investigated in future study, to clarify possible signaling pathways involved in the mentioned biofunction.

Polysaccharides also can be modified to induce corresponding changes in their structures, properties, and more important biological functions [56]. For example, when roundhead wormwood (*Artemisia sphaerocephala*) polysaccharides were sulfated using the chlorosulfuric acid/pyridine system, the resultant sulfated polysaccharides showed higher anti-tumor activity against the A549, HepG2, and Hela cells via exerting anti-proliferation effect [57]. When the hydroxyl groups of the polysaccharides isolated from the wild pepper (*Morchella angusticeps* peck) were converted to acetyl groups by a chemical esterification, it was found that the used polysaccharide acetylation induced improved immune effect and anti-inflammatory capacity in the macrophages [58]. If the polysaccharides from bast willow (*Cyclocarya paliurus*) were converted into corresponding carboxymethyl products using the chloroacetic acid and ethyl alcohol, their solubility and anti-oxidant properties were promoted [59]. In addition, a chemical phosphorylation of the asparagus (*Radix Cyathulae officinalis*) polysaccharides could yield higher anti-viral activity against the canine parvovirus [60]. In general, the target chemical selenylation is a covalent combination of polysaccharide substrates and H_2_SeO_3_ (generated from the Na_2_SeO_3_-HNO_3_ system) [61]. When the polysaccharides from alfalfa roots (*Medicago Sativa* L.) were selenylated using the Na_2_SeO_3_-HNO_3_ system, Se content of the selenylated polysaccharides was about 320 mg/kg, and the selenylated polysaccharides in the HepG2 cells showed enhanced anti-tumor and anti-oxidant effects [62]. Moreover, the obtained infrared spectrometry results showed that two new absorption peaks at respective 925 cm^−1^ and 840 cm^−1^ were observed, which indicated the corresponding C-O-Se stretching vibration and Se-O asymmetric stretching [62]. Using the Na_2_SeO_3_-HNO_3_ system, the selenylated lily polysaccharides had a very high level of Se element (39.78 g/kg) and enhanced immune function in the lymphocytes; meanwhile, the critical Se-O-C and O-Se-O bonds were detected [63]. Sharing conclusion consistence with these mentioned studies, the results of this study also confirmed that purslane polysaccharides could be selenylated by the Na_2_SeO_3_-HNO_3_ system to two relative lower selenylation extents (Se contents of 753.8 and 1325.1 mg/kg) and, subsequently, were endowed with improved immune potentials in the two immune cells. Thus, the recent results provided extra evidence for the potential application of this chemical selenylation for the interested polysaccharide modification. However, whether this selenylation also caused a change in other bioactivities for the modified polysaccharides was not investigated in this study. Thus, it is suggested that other bioactivity changes should be an interesting topic in future studies.

## 5. Conclusions

The present results highlighted that the used chemical selenylation of soluble purslane polysaccharides to the two selenylation extents could induce an enhanced immuno-modulatory effect on both macrophages and splenocytes efficiently, although the unreacted purslane polysaccharides themselves also had immune modulation in the two immune cells. Overall, the selenylated polysaccharides were more active in the macrophages to promote cell growth, phagocytic activity, and the secretion of three cytokines, including TNF-α, IL-1β, and IL-6, or more able in the mitogen-stimulated or non-stimulated splenocytes to enhance cell proliferation, as well as to elevate IFN-γ secretion but suppress IL-4 secretion. The selenylated polysaccharides also possessed a higher capacity of increasing CD4^+^/CD8^+^ ratio in the T lymphocytes. Generally, higher selenylation extent could endow the selenylated polysaccharides with higher bioactivity to the two immune cells, while higher polysaccharide doses also caused greater immune modulation in the cells. Thus, this chemical selenylation might be an applicable technique to enhance the bioactivities, such as immune modulation of natural polysaccharides. It is also suggested that other bioactivity changes of the polysaccharides arisen from the performed chemical selenylation need future investigation.

## Figures and Tables

**Table 1:** The measured cytokine levels in the macrophages incubated with PSPO, SePSPO-1, and SePSPO-2 for 24 h.

Cell Group	Dose (μg/mL)	IL-6 (pg/mL)	IL-1β (pg/mL)	TNF-α (pg/mL)
Control	None	10.69 ± 1.24 ^f^	1.55 ± 0.40 ^f^	61.39 ± 1.70 ^g^
PSPO	5	13.85 ± 1.26 ^e^	2.87 ± 0.61 ^f^	68.78 ± 1.20 ^f^
	20	33.81 ± 0.69 ^c^	5.48 ± 1.05 ^e^	112.98 ± 1.78 ^c^
SePSPO-1	5	22.30 ± 1.40 ^d^	6.29 ± 0.60 ^c^	76.76 ± 1.40 ^e^
	20	42.52 ± 0.24 ^b^	7.73 ± 0.45 ^bc^	128.96 ± 1.55 ^b^
SePSPO-2	5	32.81 ± 1.86 ^c^	8.53 ± 0.99 ^b^	86.90 ± 0.97 ^d^
	20	47.58 ± 1.61 ^a^	11.71 ± 0.77 ^a^	144.16 ± 3.49 ^a^

Different lowercase letters as the superscripts after the data in the same column suggest that the one-way ANOVA of the mean values is significantly different (*p* < 0.05).

**Table 2:** The measured T lymphocyte subpopulations in the Con A-treated splenocytes that were incubated with PSPO, SePSPO-1, and SePSPO-2 for 48 h.

Cell Group	Dose (μg/mL)	CD4^+^ (%)	CD8^+^ (%)	CD4^+^/CD8^+^ Ratio
Control	None	29.7 ± 1.2	14.4 ± 0.6	2.06 ± 0.01
PSPO	5	30.8 ± 2.0	14.8 ± 1.1	2.08 ± 0.02
	20	31.9 ± 2.8	14.5 ± 1.3	2.20 ± 0.59
SePSPO-1	5	31.9 ± 1.8	14.6 ± 0.8	2.18 ± 0.01
	20	32.3 ± 1.9	14.2 ± 0.7	2.28 ± 0.06
SePSPO-2	5	30.8 ± 0.9	13.6 ± 0.7	2.27 ± 0.08
	20	34.1 ± 3.3	14.1 ± 1.1	2.41 ± 0.09

## Data Availability

All data are contained within the article.
